# Hair Follicle Development of Rex Rabbits Is Regulated Seasonally by Wnt10b/β-Catenin, TGFβ-BMP, IGF1, and EGF Signaling Pathways

**DOI:** 10.3390/ani13233742

**Published:** 2023-12-04

**Authors:** Gongyan Liu, Ce Liu, Yin Zhang, Haitao Sun, Liping Yang, Liya Bai, Shuxia Gao

**Affiliations:** Institute of Animal Husbandry and Veterinary, Shandong Academy of Agricultural Sciences, Shandong Key Laboratory of Animal Disease Control and Breeding, Key Laboratory of Livestock and Poultry Multiomics of MARA, Jinan 251000, China; gongyanliu@foxmail.com (G.L.); liuceshiyan@163.com (C.L.); insaas@foxmail.com (Y.Z.); wwww8888@163.com (H.S.); yanglp682@163.com (L.Y.); bailiya_2005@163.com (L.B.)

**Keywords:** season, rabbit, fur quality, hair follicle development

## Abstract

**Simple Summary:**

Seasonal parameters, especially temperature and humidity, are reasonably speculated to affect livestock production performance. Among farmed animals, rabbits in particular are sensitive to extreme environmental temperature and humidity. This is not conducive to the performance of rabbit production and the improvement of product quality. Thus, a better understanding of the effects of the seasons on rabbits’ physiology is fundamental to ensuring rabbit welfare, production efficiency, and fur quality improvement. Our study proved that the skinning season has a significant influence on the fur quality and hair follicle traits of Rex rabbits. The skin quality of rabbits slaughtered in winter was the best. Seasons may regulate hair follicle development via the Wnt10b/β-catenin, TGFβ-BMP, IGF1, and EGF signaling pathways in Rex rabbits.

**Abstract:**

This experiment was conducted to study the effects of different skinning seasons on the fur quality and hair follicle development of Rex rabbits. A total of 80,150-day-old Rex rabbits were slaughtered on 15 July 2022 (summer), 15 October 2022 (autumn), 15 January 2023 (winter), and 15 April 2023 (spring) in Shandong Province (10 males and 10 females in each season). The results show that the skin weight, skin area, skin thickness, and hair follicle density of the Rex rabbits (at 150 days of age) were lower in summer than in winter (*p* < 0.05). Moreover, the coat length was shorter in summer than in spring, autumn, and winter (*p* < 0.05). The shoulder fat weight, perirenal fat weight, and perigastric fat weight of the Rex rabbits in winter were higher than those in summer (*p* < 0.05). Furthermore, the leptin levels in serum were higher in winter than in summer in the Rex rabbits (*p* < 0.05). In terms of serum biochemistry, the glucose levels were higher in autumn and winter than in spring and summer (*p* < 0.05). The cholesterol, high-density lipoprotein cholesterol (HDL), and low-density lipoprotein cholesterol (LDL) in summer had higher values than in winter in the Rex rabbits (*p* < 0.05). In winter, the expression of the *Wnt10b*, *catenin beta 1* (*CTNNB1*), *glycogen synthase kinase 3 beta* (*GSK3β*), *insulin like growth factor I* (*IGF-I*), *Type I insulin-like growth factor receptor* (*IGF-IR*), and *epidermal growth factor* (*EGF*) genes was higher (*p* < 0.05), and the expression of the *dickkopf-1* (*DDK1)*, *transforming growth factor beta 1* (*TGFβ-1*), *bone morphogenetic protein 2* (*BMP2*), and *bone morphogenetic protein 4* (*BMP4*) genes was lower than in summer (*p* < 0.05). In summer, the heat shock 70 kDa protein (HSP70) expression and CTNNB1 protein phosphorylation levels in skin tissue were higher than in spring, autumn, and winter (*p* < 0.05). In winter, Wnt10b protein expression was higher (*p* < 0.05), and GSK-3β protein phosphorylation levels were lower than in spring, autumn, and winter (*p* < 0.05). These results show that the skinning season can affect the production performance and hair follicle development of Rex rabbits. Compared with other seasons, the quality of skin from rabbits slaughtered in winter is better. Seasons may regulate hair follicle development via the Wnt10b/β-catenin, TGFβ-BMP, IGF1, and EGF signaling pathways in Rex rabbits.

## 1. Introduction

The season is an important factor affecting the growth and development of many animals, especially fur animals. Hair length and density features could affect the transfer of heat from the domestic animal’s skin to the environment [1]. The environmental temperature can affect the activities of livestock and poultry and, as a result, impact their production performance [2]. Temperature and humidity factors could affect intestinal water metabolism and cause changes in the composition of the intestinal flora [3]. Rabbit sweat glands are not well developed; this, coupled with their whole-body hair, means that they are highly sensitive to changes in the external environment’s temperature, more so than most farm animal species [4]. Rex rabbits are important fur rabbits, and the environmental temperature in different seasons seriously affects the quality of their fur. The main indicators used to evaluate the fur quality of Rex rabbits are hair density, skin area, skin weight, skin thickness, hair length, hair color, skin tensile strength, etc., [5]. Hair follicles are the tissues that produce fur in mammals, and they are one of the few organs with a periodic regeneration function after birth. According to the morphological characteristics, the hair follicle cycle is divided into anagen, catagen, and telogen [6,7,8]. Hair follicles rely on periodic cyclic growth to achieve hair growth and renewal, and their periodic changes determine the periodic growth and loss of hair, while the periodic development of hair follicles is affected by a variety of factors and is regulated by a series of signal molecules [9,10,11]. As the “material basis” of fur growth, hair follicles are not only regulated by genetic and nutritional factors but are also affected by environmental temperature and humidity [12,13,14,15]. Many studies have been conducted on animals under heat and cold stresses [16,17,18], while the effect of the seasons on fur rabbits has rarely been reported, and the mechanism of seasonal influence on hair follicle development remains unclear. In this study, Rex rabbits reared outdoors for five months were skinned in spring, summer, autumn, or winter, and their skin was removed to determine the most suitable slaughter season for Rex rabbits by assessing their fur quality, the relative expression of genes, and protein expression related to hair follicle development.

## 2. Materials and Methods

### 2.1. Experimental Design

The experiment was conducted in early January 2022, early April 2022, early July 2022, and early October 2022, and the conception and birth of Rex rabbits were ensured. Both female and young rabbits were fed the same diet formula in Tai’an City, Shandong Province, which belongs to a warm temperate continental subhumid monsoon climate area with four distinct seasons; it is suitable for an analysis of cold seasons and summer, synchronous light and temperature, and rain and heat in the same season. Spring is dry and windy (average daily temperature: 0∼9 °C), summer is hot and rainy (average daily temperature: 22∼31 °C), autumn is sunny and cool (average daily temperature: 18∼28 °C), and winter is cold and has some snow (average daily temperature: 5∼14 °C). The rabbits were reared until five months of age. In spring, summer, autumn, and winter, the rabbits had the same feeding conditions, and all of them were raised in simple outdoor cement cages with access to feed and drinking water, natural lighting, and natural ventilation; there were no cooling or insulation measures in summer and winter. The raw material composition and nutritional level of the basic diet are shown in Table 1. A total of 80,150-day-old Rex rabbits with a similar bodyweight (3428 ± 50 g) were slaughtered on 15 July 2022 (summer), 15 October 2022 (autumn), 15 January 2023 (winter), and 15 April 2023 (spring) (10 males and 10 females in each season).

### 2.2. Sample Collection and Preparation

The animals were fasted for 24h before the end of the experiment. For Rex rabbits at 150 days of age, 5 mL blood samples were placed into sterile vacuum coagulation vessels and centrifuged at 3000× *g* for 10 min after coagulation. Upper serum was collected, transferred to a sterile tube, and stored at −20 °C for the determination of blood indicators. Then, the rabbits were sacrificed via cervical dislocation. Shoulder fat, perirenal fat, and perigastric fat were carefully stripped and weighed. Mediodorsal skin samples of each rabbit sample were divided into two parts. One part was frozen in liquid nitrogen and stored in a −80 °C refrigerator for gene and protein expression detection. The other part was fixed in 4% paraformaldehyde and stored at 4 °C for subsequent hematoxylin–eosin (HE) staining for a hair follicle density analysis.

### 2.3. Determination of Indicators and Methods

#### 2.3.1. Fur Quality

At 150 days of age, the Rex rabbit skin was collected; the adhesive tissue and fat was removed, weighed, and cut in the middle of the abdomen, and then the length and width of the skin were measured using a soft ruler. The length of the skin was measured from the neck to the tail root, and the width was recorded from the narrowest part of the middle abdomen; the skin area was also calculated. The thickness of the skin was measured using vernier calipers, and the length of the coat was measured using a straightedge. The hair follicle density was observed with paraffin section and HE staining. After HE staining, photos were taken under ordinary light conditions with a microscope (Nikon, ECLIPSE 8, Tokyo, Japan), and Image-Pro Plus 6.0 analysis software was used to calculate the primary hair follicle density, secondary hair follicle density, and total hair follicle density, and the ratio of the secondary hair follicle density to the primary hair follicle density was calculated.

#### 2.3.2. Blood Indices

A rabbit insulin ELISA Kit (CSB-E06992Rb), a rabbit insulin-like growth factor 1 (IGF-1) ELISA Kit (CSB-E06994Rb), and a rabbit leptin ELISA Kit (CSB-E06971Rb) were used for the detection of serum hormone contents (CUSABIO, Wuhan, China). Serum glucose, total protein, cholesterol, triglyceride, high-density lipoprotein cholesterol (HDL), and low-density lipoprotein cholesterol (LDL) were determined using a sequential multiple analyzer (Chemray 240, Shenzhen Leidu Life Technology, Shenzhen, China).

#### 2.3.3. Total RNA Extraction and Real-Time PCR Analysis

Total RNA extraction and real-time PCR were performed as described previously [19]. The sequences of primers are shown in Table 2. Using glyceraldehyde 3-phosphate dehydrogenase (GAPDH) as the normalized gene, the mRNA relative amount of the gene was calculated using the 2 delta-delta CT method [20].

#### 2.3.4. Western Blotting

The methods for total protein extraction and sodium dodecyl sulfate–polyacrylamide gel electrophoresis (SDS-PAGE) were performed according to Liu et al. [21]. Total protein was extracted from skin tissue using a radioimmunoprecipitation (RIPA) lysis buffer (Beyotime, Shanghai, China), and the protein concentration was determined using a BCA Protein Assay Kit (CWBIO, Beijing, China). The extracted protein (50 ng/sample) was dissolved in 40 mL of SDS loading buffer (Solarbio, Beijing, China), then electrophoresed on a 12.5% SDS-page gel (Bio-Rad, Richmond, CA, USA), and transferred to a polyvinylidene fluoride (PVDF) membrane (Millipore, Billerica, MA, USA). Protein molecular mass standard markers were purchased from Thermo (Waltham, MA, USA). The membranes were blocked with 5% skim milk in PBS (Solarbio, Beijing, China) at 4 °C overnight and incubated with 1:1000 dilution primary antibodies (GAPDH, Servicebio, GB23303, Wuhan, China; HSP70, Servicebio, GB12241, Wuhan, China; Wnt10b, BIOSS, bs-3662r, Beijing, China; phospho-CTNNB1, BIOSS, bs-12854r, Beijing, China; or phospho-GSK3β antibody, Servicebio, GB114582, Wuhan, China). The membranes were then washed with Tris-buffered saline containing Tween (TBST; Solarbio, Beijing, China) and incubated with a 1:3000 dilution of a horseradish peroxidase (HRP)-conjugated goat anti-mouse IgG antibody (Beyotime, Haimen, China) at 37 °C for 1 h. The proteins were visualized using Beyo ECL reagent (Beyotime, Haimen, China). Band intensity was quantified using a Pro Plus 6.0 Bioimage Analysis System.

### 2.4. Data Processing

SPSS 26.0 statistical software was used for a one-way ANOVA analysis of the data, and Duncan’s test was used for multiple comparisons. The results are presented as the mean value and standard error of means (SEMs), and *p* < 0.05 was considered to be a significant difference.

## 3. Results

### 3.1. Fur Quality

The effects of different skinning seasons on the fur quality of Rex rabbits are shown in Table 3. As indicated, the values for skin weight, skin area, and skin thickness of the 150-day-old Rex rabbits that were skinned in summer were lower than those for the rabbits skinned in winter (*p* < 0.05). Additionally, the coat length of the Rex rabbits in summer was shorter than that in the other seasons (spring, autumn, and winter; *p* < 0.05).

### 3.2. Hair Follicle Density

**Figure 1:**
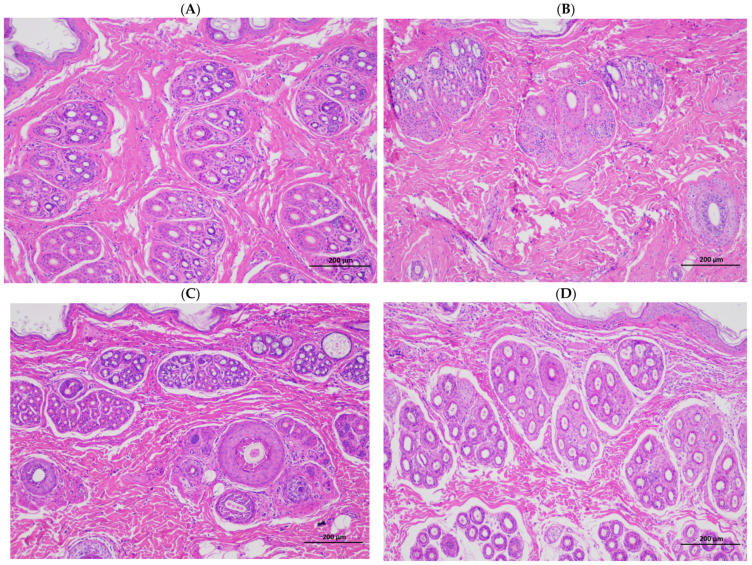
Effects of different skinning seasons on the hair follicle density of Rex rabbits; the total hair follicle density and secondary hair follicle density in Rex rabbits skinned in winter at 150 days of age were higher than those in spring, summer, and autumn seasons: (**A**) skinning in spring; (**B**) skinning in summer; (**C**) skinning in autumn; (**D**) skinning in winter. Scale bars = 200 μm.

The hair follicle density in dorsal skin was observed via paraffin sectioning and HE staining (Figure 1). The total hair follicle density and secondary hair follicle density in the Rex rabbits skinned in winter at 150 days of age were higher than those in the spring, summer, and autumn seasons (*p* < 0.05). However, the primary hair follicle density and the ratio of the secondary hair follicle density to the primary hair follicle density did not change significantly (*p* < 0.05; Table 4).

### 3.3. Fat Deposition

The fat deposition of the Rex rabbits is presented in Table 5. As shown, the shoulder fat weight, perirenal fat weight, and perigastric fat weight of the 150-day-old Rex rabbits were higher in winter than in summer (*p* < 0.05). Compared with summer, the shoulder fat weight, perirenal fat weight, and perigastric fat in the rabbits skinned in winter increased by 28.81%, 62.90%, and 41.54%, respectively.

### 3.4. Serum Hormones and Biochemical Indices

The serum hormones and biochemical indices of the Rex rabbits are presented in Table 6. As indicated, the leptin levels in the serum of the Rex rabbits skinned in winter at 150 days of age were higher than in the rabbits skinned in summer (*p* < 0.05), and there was no significant difference between spring, summer, autumn, and winter skinning in terms of serum hormones (insulin and IGF-I) (*p* > 0.05). For serum biochemistry, the glucose levels in the Rex rabbits skinned in autumn and winter were higher than in those skinned in spring and summer (*p* < 0.05). The cholesterol, HDL, and LDL in the Rex rabbits skinned in summer at 150 days of age were higher than in those skinned in winter (*p* < 0.05).

### 3.5. Gene Expression of Hair Follicle Development

The effects of the different skinning seasons on the gene expression of hair follicle development in the Rex rabbits are shown in Table 7. The expression of the *Wnt10b*, *CTNNB1*, *GSK3β*, *IGF-I*, *IGF-IR,* and *EGF* genes in the skin tissue of the Rex rabbits at 150 days of age was higher for winter skinning than for summer skinning (*p* < 0.05), and the expression of the *DDK1*, *TGFβ-1*, *BMP2*, and *BMP4* genes was lower for winter skinning than for summer skinning (*p* < 0.05).

### 3.6. Protein Expression and Phosphorylation Level

**Figure 2:**
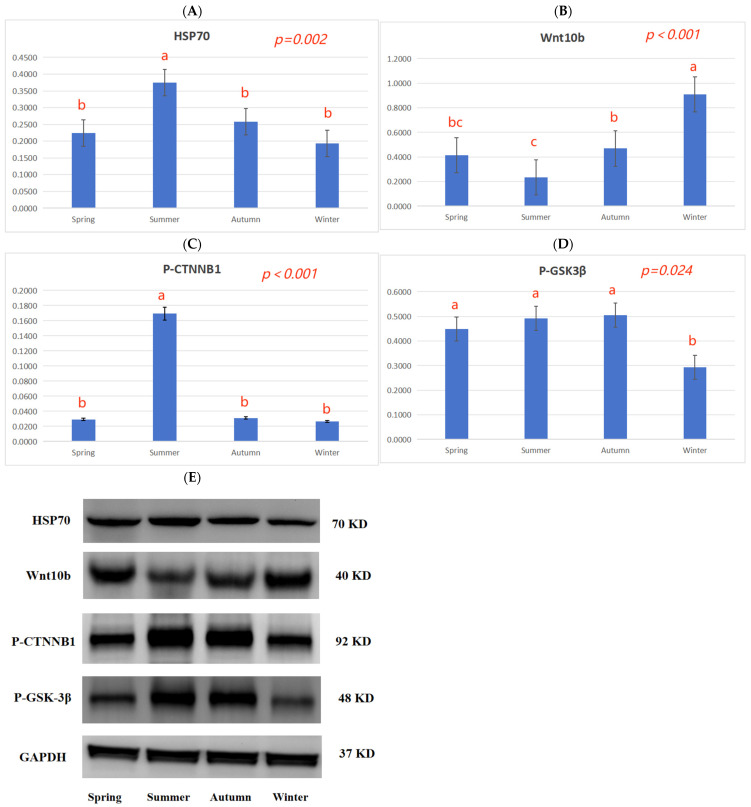
Effects of different skinning seasons on the protein expression of hair follicle development in Rex rabbits. (**A**) HSP70 protein expression in skin tissue; (**B**) Wnt10b protein expression in skin tissue; (**C**) CTNNB1 protein phosphorylation levels in skin tissue; (**D**) GSK-3β protein phosphorylation levels in skin tissue; (**E**) blotting strip. Data are expressed as the mean and standard error of means (SEMs), n = 8, and different letters in the same row denote a significant effect (*p* < 0.05).

The effects of different skinning seasons on the protein expression (HSP70 and Wnt10b) and protein phosphorylation (CTNNB1 and GSK-3β) levels of hair follicle development in the Rex rabbits at 150 days of age are shown in Figure 2. In summer, the HSP70 protein expression and CTNNB1 protein phosphorylation levels in the skin tissue of the Rex rabbits were higher than in spring, autumn, and winter (*p* < 0.05; Figure 2A,C). In winter, Wnt10b protein expression was higher than that in spring, autumn, and winter (*p* < 0.05; Figure 2B), and GSK-3β protein phosphorylation levels were lower than in the other seasons (spring, autumn, and winter, *p* < 0.05; Figure 2D).

## 4. Discussion

Global warming and abnormally high ambient temperatures have adversely affected the livestock sector in recent decades [22]. In China, where the temperature changes in different seasons, rabbits in particular are sensitive to extreme environmental temperature and humidity. This is not conducive to the performance of rabbit production and the improvement of product quality. Compared with the summer, the shoulder fat weight, perirenal fat weight, and perigastric fat increased in winter by 28.81%, 62.90%, and 41.54%, respectively (Table 5). The observed differences are consistent with those in previous research reports [23,24,25,26]. Ambient temperatures affect the lipid metabolism of broilers, and high cholesterol and triglyceride contents in the blood reflect enhanced lipid catabolism [27]. Leptin is a hormone secreted by adipose tissue; it is involved in promoting fat breakdown and utilization, and its content in serum is proportional to the amount of adipose tissue in the animal [28,29,30]. Leptin acts on receptors located in the central nervous system to regulate the behavior and metabolism of organisms [31], and it regulates the energy balance and weight of organisms via a negative feedback mechanism [32]. The HDL receptor mediates the selective uptake of cholesterol [33], and heat stress may reduce the activity of glucose-6-phosphate dehydrogenase, thus reducing the activity of nicotinamide adenine dinucleotide dehydrogenase and inhibiting energy metabolism [34]. In this study, cholesterol, HDL, and LDL were higher in summer than in winter in the Rex rabbits (Table 6), suggesting that the season can affect lipid metabolism and energy balance.

Rabbits with fur have poor heat dissipation mechanisms and are more easily affected by heat stress, and this reduces their performance and the economic efficiency of farms. The quality of fur is mainly based on the hair density, hair length, skin area, and skin thickness, and the density of the hair follicle is a particularly important indicator for evaluating the quality of Rex rabbits’ skin. The skin in summer is generally thin and breaks easily, and the wool can also fall off easily, which affects the rabbit’s skin quality. Compared with winter, the present results show that skinning in summer decreased the skin weight, skin area, skin thickness (Table 3), and hair follicle density of the rabbits (Table 4; Figure 1). This finding is consistent with previous experimental results, which showed that heat stress can reduce the hair length and hair follicle density of Rex rabbits [35]. The expression of several heat shock proteins is increased during summer; this helps maintain cellular redox homeostasis to ensure cell survival. In particular, HSP70 is highly expressed and is a key marker of heat stress in hair follicle tissue [36,37,38]. In summer, the HSP70 protein expression in skin tissue was higher than in spring, autumn, and winter (Figure 2A), and the fur quality was poor (Table 3); this is closely related to the high temperatures experienced in summer.

The Wnt family is a class of secreted lipid-modified glycoproteins, which play an important regulatory role in the development of animal hair follicles [39]. The classical Wnt signaling pathway, composed of the Wnt protein, β-catenin, frizzled-4, and the adenomatous polyposis coli complex, is involved in skin biological processes [40]. Wnt10b promotes hair follicle growth and dermal papilla cell proliferation via the Wnt/β-catenin signaling pathway in Rex rabbits [41]. Wnt signaling requires the sequestration of glycogen synthase kinase 3 (GSK3β) inside multivesicular endosomes [42]. Dickkopf-associated protein 1 (DKK1) is an important antagonist of the Wnt signaling pathway. The continuous overexpression of DKK1 during the embryonic development stage prevents the formation of hair follicles in animals, and the overexpression of DKK1 after birth inhibits the growth of hair follicles. DKK1 can inhibit the Wnt signaling pathway by inhibiting β-catenin activity, leading to hair follicle degeneration [43]. In winter, *Wnt10b*, *CTNNB1*, and *GSK3β* gene expression was higher than in summer, and the *DDK1* gene expression was lower than in summer (Table 7), indicating that high temperatures could inhibit hair follicle development by inhibiting the Wnt10b/β-catenin signal.

The bone morphogenetic protein (BMP) signaling pathway plays an important role in controlling the growth and development of animal skin hair follicles [44]. BMP signals are conducive to the maintenance of dermal papilla activity; BMP2 and BMP4 participate in the formation of the hair follicle matrix, and the expression levels of BMP2 and BMP4 during the growth phase are lower than during the resting phase, inhibiting the transformation of hair follicles from the resting phase to the growth phase [45]. After the overexpression of BMP2, cell proliferation and differentiation between hair follicle stem cells and dermal papilla cease [46]. BMP signaling inhibits Wnt signaling by regulating β-catenin phosphorylation. Transforming growth factor-β1 (TGF-β1) is expressed in the inner root sheath during the transition from the growth stage to the degeneration stage, which can cause hair follicles to transition from the growth stage to the degeneration stage and inhibit cell proliferation [47]. In winter, the expression of the *TGFβ-1*, *BMP2*, and *BMP4* genes was lower than that in summer (Table 7), and the CTNNB1 protein phosphorylation levels in the skin tissue in summer were higher than in spring, autumn, and winter (Figure 2C), indicating that the seasons may regulate hair follicle development via the TGF-β/BMP signaling pathway.

IGF1 has a positive effect on hair follicle development; it is expressed in both the hair follicles and dermal fibroblasts of rats, participates in apoptosis, and promotes the proliferation of dermal cells [48]. In addition, EGF stimulates hair follicle elongation and maintains hair follicle growth [49], and a subcutaneous injection of EGF in Angora rabbits can increase the growth of hair follicles and induce hair follicle regeneration [50]. Knockdown of the EGF receptor gene leads to a decrease in hair follicle formation and facilitates the transition of hair follicles from anagen to catagen, which is in line with previous findings [51]. The expression of *IGF-I*, *IGF-IR*, and *EGF* in the rabbits skinned in summer was lower than that in the rabbits skinned in winter (Table 7), which suggests that IGF1 and EGF signaling may be the key target signals in temperature-regulated hair follicle development in Rex rabbits.

## 5. Conclusions

The skinning season can affect the production performance and hair follicle development of Rex rabbits. The skin quality of Rex rabbits slaughtered in winter is better than in the other seasons. Seasons may regulate hair follicle development via the Wnt10b/β-catenin, TGFβ-BMP, IGF1, and EGF signaling pathways in Rex rabbits.

## Figures and Tables

**Table 1:** Composition and nutrient levels of basal diets (air-dry basis).

Raw Material Composition	Content (%)	Nutrient Levels ^2^	Content (%)
Corn	5.0	Digestible energy (MJ/kg)	10.23
Soybean meal	8.0	Dry matter	89.82
Barley	6.0	Crude protein	16.12
Wheat bran	15.0	Ether extract	2.80
Corn germ meal	16.0	Crude fiber	17.38
Corn husk	17.0	Neutral detergent fiber	38.74
Alfalfa meal	15.0	Acid detergent fiber	23.08
Soybean straw powder	7.0	Acid detergent lignin	6.29
Rice hull powder	8.0	Crude ash	9.03
Calcium hydrogen phosphate	1.5	Calcium	0.95
Sodium chloride	0.5	Total phosphorus	0.45
Premix ^1^	1.0	Lysine	0.60
Total	100.0	Methionine	0.65

^1^ Premix provided the following per kg of diets: vitamin A 8 000, IU; vitamin D3, 1500 IU; vitamin E, 45 mg; vitamin K3, 2.0 mg; vitamin B1, 1.0 mg; vitamin B2, 3.0 mg; vitamin B6, 1.5 mg; nicotinic acid, 30 mg; pantothenic acid, 50 mg; folic acid, 0.5 mg; choline chloride, 100 mg; iron, 50 mg; copper, 10 mg; zinc, 50 mg; manganese, 10 mg; iodide, 0.5 mg; selenium, 0.05 mg; lysine, 1.5 g; methionine, 0.5 g; the rest was Maifan stone carrier complement. ^2^ Digestible energy was a calculated value, while the others were measured values.

**Table 2:** Information on primers.

Gene	Accession Number	Primer Sequence (5′-3′)	Product Length, bp
*GAPDH*	NM_001082253.1	F: TTCCAGTATGATTCCACCCACG	232
		R: GGGCTGAGATGATGACCCTTTT	
*Wnt10b*	XM_002711076.4	F: GGCGAGAATGAGAATCCATAACAA	196
		R: GTTGTGGGTGTCAATGAAGATGG	
*CTNNB1*	XM_051852655.1	F: TGGATACCTCCCAAGTCCTGTA	207
		R: CCAGACGCTGAACATTAGTAGGAT	
*GSK3β*	XM_017347066.1	F: TGAGGTCTATCTTAATCTGGTGCTG	183
		R: TGTGGTTTAATATCCCGATGGC	
*DDK1*	NM_001082737.2	F: ATGGGTATTCCCGCAGAACC	150
		R: CCTTGAGGACGGGCTTACAG	
*TGFβ-1*	XM_008249704.2	F: CTGCTGTGGCTCCTAGTGTTGA	134
		R: AGCCGCAGTTTGGACAGGAT	
*BMP2*	NM_001082650.1	F: GGTGGAACGACTGGATTGTG	146
		R: CGGAATCTTAGAGTTCACGGAGT	
*BMP4*	NM_001195723.1	F: AGGGACCAGCGAAAACTCTG	149
		R: TGTTTATCCGGTGGAAGCCC	
*IGF-I*	NM_001082026.1	F: TCTCTTCTACCTGGCCCTCTG	155
		R: TGCTGGAGCCGTATCCTGT	
*IGF-IR*	XM_008248786.2	F: ACGTGGAAGAACCGCATCAT	129
		R: GTCCTGCCCATCATACTCCG	
*EGF*	XM_017347349.1	F: CACTGCTCAGAAGGCTACCAA	184
		R: GAAATGGCGGAACAGAATCAG	

*GAPDH*: glyceraldehyde-3-phosphate dehydrogenase; *CTNNB1*: catenin beta 1; *GSK-3β*: glycogen synthase kinase 3 beta; *DKK1*: dickkopf-1; *TGF-β1*: transforming growth factor beta 1; *BMP2*: bone morphogenetic protein 2; *BMP4*: bone morphogenetic protein 4; *IGF-I*: insulin like growth factor I; *IGF-IR*: type I insulin-like growth factor receptor; *EGF*: epidermal growth factor; F: forward primer; R: reverse primer.

**Table 3:** Effects of different skinning seasons on the fur quality of Rex rabbits.

Items	Spring	Summer	Autumn	Winter	SEM	*p*-Value
Skin weight (g)	499.05 ^b^	482.37 ^b^	542.25 ^a^	558.10 ^a^	7.059	<0.001
Skin area (cm^2^)	1818.15 ^b^	1667.15 ^c^	1961.05 ^a^	1985.40 ^a^	26.640	<0.001
Skin thickness (mm)	8.66 ^a^	8.03 ^c^	8.40 ^b^	8.80 ^a^	0.053	<0.001
Coat length (cm)	1.86 ^a^	1.56 ^d^	1.64 ^c^	1.78 ^b^	0.015	<0.001

Data are expressed as the mean and standard error of means (SEMs), n = 20, and different letters in the same row denote a significant effect (*p* < 0.05).

**Table 4:** Effects of different skinning seasons on the hair follicle density of Rex rabbits.

Items	Spring	Summer	Autumn	Winter	SEM	*p*-Value
Total hair follicle density (count/mm^2^)	347.13 ^b^	292.01 ^b^	340.58 ^b^	366.68 ^a^	5.586	<0.001
Primary hair follicle density (count/mm^2^)	13.69	12.99	13.69	13.46	0.320	0.861
Secondary hair follicle density (count/mm^2^)	333.44 ^b^	279.02 ^c^	326.88 ^b^	353.22 ^a^	5.532	<0.001
Secondary hair follicle/Primary hair follicle ratio	24.81	22.04	24.11	26.48	0.611	0.071

Data are expressed as the mean and standard error of means (SEMs), n = 8, and different letters in the same row denote a significant effect (*p* < 0.05).

**Table 5:** Effects of different skinning seasons on the fat deposition of Rex rabbits.

Items	Spring	Summer	Autumn	Winter	SEM	*p*-Value
Shoulder fat weight (g)	14.68 ^b^	12.98 ^b^	12.74 ^b^	18.42 ^a^	0.588	0.001
Perirenal fat weight (g)	57.29 ^b^	53.05 ^b^	60.66 ^b^	86.42 ^a^	0.965	0.006
Perigastric fat weight (g)	18.09 ^ab^	15.96 ^b^	17.54 ^ab^	22.59 ^a^	1.783	0.046

Data are expressed as the mean and standard error of means (SEMs), n = 8, and different letters in the same row denote a significant effect (*p* < 0.05).

**Table 6:** Effects of different skinning seasons on the serum hormones and biochemical indices of Rex rabbits.

Items	Spring	Summer	Autumn	Winter	SEM	*p*-Value
Serum hormones						
Insulin (mmol/mL)	2.79	2.12	1.65	3.59	0.395	0.343
IGF-I (ng/mL)	0.56	0.53	0.48	0.50	0.019	0.539
Leptin (ng/mL)	12.62 ^ab^	9.72 ^b^	12.87 ^ab^	15.05 ^a^	0.299	0.028
Serum biochemical						
Glucose (mmol/L)	4.77 ^b^	5.10 ^b^	9.88 ^a^	8.83 ^a^	0.464	<0.001
Total protein (g/L)	56.49	58.89	60.55	59.43	0.845	0.394
Triglyceride (mmol/L)	0.70	0.82	0.72	0.37	0.063	0.057
Cholesterol (mmol/L)	1.10 ^a^	1.33 ^a^	1.09 ^a^	0.66 ^b^	0.070	0.003
High-density lipoprotein (HDL, mmol/L)	0.70 ^a^	0.76 ^a^	0.71 ^a^	0.30 ^b^	0.057	0.008
Low-density lipoprotein (LDL, mmol/L)	0.23 ^b^	0.41 ^a^	0.22 ^b^	0.17 ^b^	0.029	0.013

Data are expressed as the mean and standard error of means (SEMs), n = 8, and different letters in the same row denote a significant effect (*p* < 0.05).

**Table 7:** Effects of different skinning seasons on the gene expression of hair follicle development in Rex rabbits.

Items	Spring	Summer	Autumn	Winter	SEM	*p*-Value
*Wnt10b*	0.28 ^b^	0.22 ^b^	0.21 ^b^	0.46 ^a^	0.025	0.038
*CTNNB1*	1.05 ^a^	0.56 ^b^	0.90 ^a^	1.30 ^a^	0.142	0.336
*GSK3β*	3.42 ^a^	1.62 ^b^	2.21 ^ab^	4.68 ^a^	0.304	0.039
*DDK1*	0.47 ^b^	0.96 ^a^	0.68 ^ab^	0.26 ^b^	0.082	0.013
*TGFβ-1*	0.74 ^b^	1.67 ^a^	1.27 ^ab^	0.68 ^b^	0.149	0.048
*BMP2*	0.66 ^b^	1.58 ^a^	1.12 ^ab^	0.70 ^b^	0.133	0.039
*BMP4*	0.59 ^ab^	1.31 ^a^	1.17 ^ab^	0.56 ^b^	0.028	0.049
*IGF-I*	0.81 ^ab^	0.34 ^b^	0.23 ^b^	1.28 ^a^	0.120	0.003
*IGF-IR*	24.06 ^ab^	6.93 ^b^	28.84 ^ab^	34.74 ^a^	15.495	0.015
*EGF*	2.25 ^ab^	0.95 ^b^	1.44 ^b^	3.97 ^a^	0.357	0.009

*CTNNB1*: catenin beta 1; *GSK-3β*: glycogen synthase kinase 3 beta; DKK1: dickkopf-1; *TGF-β1*: transforming growth factor beta 1; *BMP2*: bone morphogenetic protein 2; *BMP4*: bone morphogenetic protein 4; *IGF-I*: insulin like growth factor I; *IGF-IR*: type I insulin-like growth factor receptor; *EGF*: epidermal growth factor. Data are expressed as the mean and standard error of means (SEMs), n = 8, and different letters in the same row denote a significant effect (*p* < 0.05).

## Data Availability

The data that support the findings of this study are available from the corresponding author upon reasonable request.
